# Report of a Few Interesting Surgical Cases with Unusual Findings

**Published:** 1916-12

**Authors:** C. W. Roberts

**Affiliations:** Atlanta, Ga.


### REPORT OF A FEW INTERESTING SURGICAL CASES WITH UNUSUAL FINDINGS.

By C. W. Roberts, M. D., Atlanta, Ga.

1. Ruptured Intestine, Strangulated Hernia, Peritonitis.
2.. Strangulated Inguinal Hernia, Left Side.
3. Appendix in Fork of Abdominal Aorta.
4. Caries of Spine.

The following cases observed in my service at the Douglas Hospital Douglas, Ga., are brought to your attention, being deemed worthy of special consideration for two reasons: first, the confusing of symptoms presented, making satisfactory diagnosis previous to operation impossible; second, the unusual and interesting findings when subjected to operative measures.

**Case 1**.—Patient, white male, age 58, admitted to Hospital October 23, 1911, complaining of having been kicked in the left lower quadrant of the abdomen by a horse, some 48 hours previous to admittance. At time of injury patient had no symptoms whatever, and, as far as he knew, was well and strong, barring general debility incident to beginning old age. Following injury patient walked some distance to his residence, little disturbed, and later summoned his physician· A cursory examinations failed to show any gross lesions, consequently patient was put to bed to await result. The following day it was noted that the abdomen was tense, tender to palpating hand, vomiting ensued, pulse was accelerated and considerable pain experienced, with rise in temperature. More careful examination revealed a small lump, size of hen's egg, below Poupart's ligament in right groin. No previous history of abnormalities in this region could be determined; had never been noted previously. These findings brought patient to hospital to seek relief. When first seen at hospital patient presented very typical symptom of peritoneal inflammation with ordinary systemic reaction. Eyes were sunken, face expressive of grave lesions. In the right groin, a tender, somewhat moveable, irreducible, non-fluctuating lump, size of hen's egg, was noted. This, with abdominal distress and distention, coupled with former negative history, suggested immediate operation. Accordingly patient was anesthetized, an incision made over lump in groin, tissues dissected off until the peritoneal pouch was reached. This was now opened, and found to contain a mass of omontum only, but a large quantity of flocculent fluid escaped from the peritoneal cavity, with occasional masses of fresh exudate. The omental mass was pushed back into the abdomen and the hernial opening closed. Now, the abdomen was opened over seat of injury in left lower quadrant. The tissues of abdominal wall were found severely bruised, very much thickened and stuck to underlying viscera. These having been released, and the intestines exposed, a hole size of a dime came into view. Large quantities of fecal stained fluid and pus were evacuated. The hole in bowel was closed, drainage tube inserted and abdomen closed around it. A gauze wick was also left down on opening in peritoneum, in the groin. Patient made very satisfactory and unsuspected recovery. The interpretation set on this case is that his femoral hernia descended as a result of increased intra-abdominal pressure incident to kick, which also ruptured the gut, causing general peritonitis.

**Case II.**—A colored man, age 46, brought to hospital November 2, 1910, suffering from strangulated hernia of left side of fourteen hours' duration. On evening previous to admittance, while going up stairs to retire, patient felt hernia descend. On many occasions before reduction had been accomplished by himself without difficulty. Having failed in this instance, a physician was called, who manipulated the protruding mass for several hours, failing finally to replace it. Immediately patient was sent to hospital. Examination on admittance showed large mass, size of the doubled fist, protruding from the left external inquinal ring into scrotum. History of old rupture with usual signs easily made the diagnosis· Taxis was instituted, but proved of no avail. Gurgling could be heard in the protruding viscera. Immediate operation was performed as follows: A large incision corresponding in direction and immediately above the left inguinal canal was made, exposing the external ring. The peritoneal pouch was now isolated. It was found to contain the cecum and appendix, with a few coils of ileum. The appendix was acutely inflamed and studded with little wart-like lumps, giving appearance of tubercular involvement. The appendix was constricted by the external abdominal ring. Appendectomy performed, and the bowel returned into abdomen. Investigation before releasing the cecum showed that the bowel came from its normal position, to which it easily returned when released. The hernia was closed; patient made an uneventful recovery.

**Case III.**—A white woman, age 26, entered hospital November 12, 1913, complaining of abdominal pain. The following history was given: During the past thirty months eleven attacks of acute pain in right side have been experienced. Attacks were accompanied by nausea, but at only two of them had she vomited. Bowels have grown progressively constipated since first attack. With first attack no unusual symptoms were noted, but in last three patient had been forced to pass water many times during and for few days following attacks. Pulse and temperature increase have been noted in last two attacks.· No blood has been passed in urine, stools have been normal. Patient gives positive history of tuberculosis in family, no history of malignancy. Some twelve or eighteen pounds have been lost in the past twelve months. Weakness with tingling sensation of right leg is complained of. Intense soreness follows attacks, the patient finding it necessary to stay in bed for a few days following each seizure. For past twelve months symptoms have been present more or less all the time, more exaggerated at times—i.e·, following exertion etc., The character of pain is described as sharp, cutting, lancinating. Attacks have all, with possible exception of one or two, come on suddenly. Examination on admittance shows following: Healthy-looking woman, well nourished, florid skin. Right leg is drawn up continuously. Entire right abdomen tender, very rigid. Pulse and temperature normal. Leucocyte count—various counts—30,000 polymorpho-nuclear cells. Right kidney is freely movable, slightly enlarged, but not very tender. No increased resistance of abdominal wall or pain, felt on palpation over appendix. Pelvic organs were negative, repeated urine examinations negative. No abnormality noted in lumbar or dorsal spine. Examination from day to day showed symptoms and signs the same, with possibly a little localization of stiffness of abdominal wall, to an area in mid line, immediately below umbilicus. There was no history of injury. Patient was subjected to Laparotomy on the persistence of a high leucocyte count. Patient at no time appeared sick.

The right rectus incision was chosen. The abdomen opened, a large mass apparently post-peritoneal, was discovered lying on and at the bifurcation of abdominal aorta. The cecum was now searched for, and found drawn well over in mid-line, covered and tied up in mass referred to. Adhesions were carefully dissected away, the large bowel (descending colon and cecum) lifted up, when the appendix was found, post-ceceal, lying in an abscess cavity, which surrounding the great vessels. The appendix was tied off, cavity carefully sponged out and abdomen closed, save tract for purpose of drainage· Patient made perfect recovery after operation. A review of the history, signs and symptoms of this case will immediately suggest to our minds any one of a number of surgical affections of the right side; although proving finally to be the most common, that of appendical inflammation, the symptoms were so atypical as to make differential diagnosis impossible. Of especial interest was the existence of a persistent high leucocytosis without temperature or pulse reaction, to be explained probably by the proximity of the abscess cavity to the large vessels and lymph channels, and also the unusual location of cecum and appendix, showing that the appendix may occupy very freakish positions in the abdomen.

**Case IV.**—That of a young man, age 28, admitted October 28, 1914, with following history: Health previous to the past two years was good, there being no disturbance worthy of mention. Trouble began by attacks of vomiting, which came on usually in the morning, but occasionally in afternoon. Pain was not felt at beginning. Having been a rather excessive drinker, his physician explained vomiting on this basis. Alcoholics were, therefore, given up with ease, but symptoms continued. Weight was gradually and slowly lost. Patient was easily fatigued. Was very nervous, restless, and appetite failed. Attacks of vomiting later became associated with epigastric and general abdominal pain of severe and agonizing character. No blood, however, was even noticed in the vomitus. Periods between attacks were remarkably free from symptoms, no pain, no tenderness, good digestion, never any jaundice.

Constipation was mentioned as a rather persistent symptom· Condition thus went on, patient enjoying what he described as good health, except during attacks. These grew more and more severe, came on without warning, suddenly and as follows: Vomiting would ensue, then pain located in mid-line just above umbilicus, vomiting would continue without easing for from three to five days—first contents of stomach, then a straw colored fluid, later bile stained. No food could be taken. There was no temperature or pulse reaction. A little tenderness, not marked over gall-bladder. Presently symptoms would subside, and then patient would immediately feel well. These attacks brought him to hospital for relief. On admittance patient is observed to be poorly nourished, rather anemic, overworn individual, who gets about with rather guarded step. General examination shows very little; abdomen, gall-bladder, stomach, urine, blood, temperature and pulse all about normal. An angulation in spine at junction of dorsal and lumber vertebrae is noted. The reflexes of the lower extremities are markedly exaggerated. Being retained a few days for observation, an attack as described above was experienced. There was nothing significant in stools following the attack, no blood in vomitus, no systematic reaction. Stomach analysis showed simple hyperacidity. Based upon the periodicity of symptoms, the absence of hematemesis and the presence of severe pain, a diagnosis of gall-bladder inflammation was made, being confirmed by every physician who had been in attendance when attack was experienced. Consequently the abdomen was opened above umbilicus with following findings:

Stomach and Pylorus normal. There was no apparent abnormality in the appearance or feel of the Duodenum. No stones could be detected in the gall-bladder or ducts. A few adhesions were noted passing from common duct on to duodenum and partially constricting it. These were relieved, with the hope of giving relief, it being thought that a vicious circle was thus created. Abnomen was now closed. Everything went well for five days, when suddenly a most typical attack was again experienced. Attention was now directed to back, because of the markedly exaggerated reflexes which were still present coupled with the visible angulation in dorsal spine. A plaster jacket was applied, hyperextending spine and supporting shoulders. Reflexes rapidly cleared up, bowels became regular, appetite improved, and patient was discharged. After several months, patient reports that he has had no further attacks.

The above case, apparently relieved, with another of similar symptoms, operated upon at another hospital, for some supposed abdominal trouble without improvement and later admitted here, a diagnosis of Pott's disease made, treatment instituted, later discharged free from symptoms, and still another operated upon five times, three times in this hospital and twice elsewhere, for symptoms pointing to kidney, gall-bladder, stone or appendical inflammation and finally, without relief, later discovered to be tuberculosis of spine, proper fixation apparatus applied and discharged, symptoms relieved,—is sufficient to make it of pressing importance to exclude Caries of the Spine, when dealing with all forms of abdominal pain.

828 Candler Building.

